# Maximum Strength, Rate of Force Development, Jump Height, and Peak Power Alterations in Weightlifters across Five Months of Training

**DOI:** 10.3390/sports5040078

**Published:** 2017-10-13

**Authors:** W. Guy Hornsby, Jeremy A. Gentles, Christopher J. MacDonald, Satoshi Mizuguchi, Michael W. Ramsey, Michael H. Stone

**Affiliations:** 1 Department of Coaching and Teaching Studies, West Virginia University, 375 Birch Street, Morgantown, WV 26505, USA; 2 Department of Sport, Exercise, Recreation, and Kinesiology, East Tennessee State University, 1276 Gilbreath Drive, Johnson City, TN 37614, USA; jeremygentles@gmail.com (J.A.G); harahara10@hotmail.com (S.M.); RAMSEYM@mail.etsu.edu (M.W.R.); stonem@etsu.edu (M.H.S.); 3 Department of Kinesiology, Recreation, and Sport Studies, Coastal Carolina University, Williams-Brice 111, Conway, SC 29526, USA; cmacdonal@coastal.edu

**Keywords:** weightlifters, block periodization, athlete monitoring, rate of force development, peak force, vertical jump, isometric mid-thigh pull

## Abstract

The purpose of this monitoring study was to investigate how alterations in training affect changes in force-related characteristics and weightlifting performance. Subjects: Seven competitive weightlifters participated in the study. Methods: The weightlifters performed a block style periodized plan across 20 weeks. Force plate data from the isometric mid-thigh pull and static jumps with 0 kg, 11 kg, and 20 kg were collected near the end of each training block (weeks 1, 6, 10, 13, 17, and 20). Weightlifting performance was measured at weeks 0, 7, 11, and 20. Results: Very strong correlations were noted between weightlifting performances and isometric rate of force development (RFD), isometric peak force (PF), peak power (PP), and jump height (JH). Men responded in a more predictable manner than the women. During periods of higher training volume, RFD was depressed to a greater extent than PF. JH at 20 kg responded in a manner reflecting the expected fatigue response more so than JH at 0 kg and 11 kg. Conclusions: PF appears to have been more resistant to volume alterations than RFD and JH at 20 kg. RFD and JH at 20 kg appear to be superior monitoring metrics due to their “sensitivity.”

## 1. Introduction

Managing the overall training process of competitive athletes is a complex and sometimes daunting challenge. A common coaching strategy is to break down the calendar into smaller, more manageable periods of time, allowing for specific training-adaptation objectives to be targeted [1,2,3,4]. An athlete monitoring system can provide the coach with invaluable data concerning athlete preparation and preparedness. While completely accurate predictions of an athlete’s response to a given training stimuli may not be possible, [5,6] the general direction of the adaptation process can be predicted based on the training prescription and the manner in which training stress is being directed [5,6]. Appropriate monitoring can provide the coach with quantitative information that allow comparisons to be made between the theoretically based, pre-determined expectations, and the actual results of the training prescription. Conceptually, this describes the benefits of a detailed, retrospective analysis of the training process.

A primary objective of an applied sport scientist is to quantify input and output factors (e.g., training stimuli, accumulative fatigue levels, etc.) that affect an athlete’s performance. Qualitative performance outcomes relate to the fitness-fatigue paradigm and the level of athlete “preparedness”, and thus can provide an estimate of athletes’ potential to perform well [5,6]. Quantitative measures deal with the magnitude of specific adaptations, as well as actual performance outcomes. Although long-term athlete monitoring is still in its infancy, a comprehensive athlete monitoring system can provide a framework for the creation of a sport science-coach feedback system, by which evidence-based adjustments to training can be made [5,6,7].

### 1.1. Background and Nuances

While certainly beneficial, detailed athlete monitoring is quite difficult, particularly with advanced athletes, as alterations in physiology and performance are often quite subtle. Indeed, relatively long-term studies (>12 weeks) of strength-power athletes, particularly high-level weightlifters, is nearly non-existent. Furthermore, these subtle alterations must be communicated to the athlete and coach in a manner which allow the objectives to be met and appropriate (if necessary) training alterations to be made. This information can be communicated to the coaches as group data (and as individual athlete data—not discussed here). Thus, this study was undertaken in a non-traditional manner with these subtleties in mind.

Many problems dealing with longitudinal training studies in well-trained athletes have been reported in the literature. For example: (1) the duration of studies are not long enough to produce or at least detect important aspects of adaptation [6,8]; (2) failure to report training workloads executed in or out of the weight room [9,10]; (3) failure to use monitoring tools with sufficient sensitivity to detect specific adaptions [7] and; (4) the experiment environment does not closely enough match an athletes “real world” experience [6,8]. Indeed, maintaining ecological validity was a paramount focus of the present study.

Periodization is an inclusive theoretical paradigm that coaches use to direct training adaptations toward enhancing athletes’ performance capabilities, in order to accomplish competitive goals [1,2]. Modern Periodization involves breaking the training plan into smaller, more manageable periods (fitness phases and time frames), or “blocks”, and allows for the responses of those sequenced blocks to converge over time [1,2]. The primary goals of periodization are: (1) fatigue management and reduction of the overtraining potential and; (2) manipulating performance in a manner to achieve peak performance at the right time or provide maintenance [1,2]. Programming (sets, repetitions, exercises etc.) gives fitness phases structure and is the means whereby targeted fitness characteristic(s) and fatigue management can be achieved. Block periodization schemes, particularly among athletes [1,2,11], can provide superior adaptive efficacy and training efficiency. Block periodization depends upon “stages”, each containing three fitness phases [1,2]: Accumulation, Transmutation, and Realization. In general, for accumulation, an emphasis is placed on higher volume and less specific training is conducted that emphasizes alterations in aspects such as work capacity, body composition, and basic strength. Transmutation involves somewhat more specific exercises with lower volume and somewhat higher intensities of training, and can entail large increases in maximum strength for specific exercises. Realization typically deals with very specific exercises that are generally power-oriented for strength-power athletes, and typically involves a taper to reduce accumulated fatigue. Often, a planned over-reaching phase is used in conjunction with the taper [1,2,12]. For strength-power sports during accumulation, the emphasis would generally be on strength endurance, work capacity, and body composition alterations, particularly total muscle cross-sectional area (CSA) and the muscle fiber type II/I CSA. Transmutation would be programmed to emphasize exercise specific strength gains and further target the II/I CSA area. Realization would involve an emphasis on increasing task specific power output, as well as a taper, in order to dissipate fatigue and possibly alter myosin heavy chain type from IIa back toward IIx [1,2]. Blocks can be manipulated to emphasize compatible combinations of exercises with emphasis on one or more fitness characteristics [1,2,3,4]. For example, blocks could contain exercises and loading schemes for both strength and power, but one or the other may have a greater emphasis or training focus (e.g., strength/power or strength/power), depending upon factors such as exercise selection, loading, and velocity of movement. 

Assessment of monitoring studies requires a theoretical understanding of the training process to allow for comparisons between the expected adaptation(s) and the actual adaptation(s) (see the expected general adaptation trend). Many of these expectations are based on the adaptation focus of a given training phase (emphasis/de-emphasis) and the amount of accumulated fatigue present [1,2]. Additionally, consideration should be given to the order in which the training phases are sequenced [1,2].

### 1.2. Brief Weightlifting Overview 

While basic technique is quite similar [13], more successful weightlifters are typically stronger and more powerful than less accomplished weightlifters [14]. Indeed, weightlifting performance depends on the ability of the lifter to accelerate the barbell within a critical time [13]. The time allowed to complete a snatch or clean and jerk is less than the time necessary to produce peak force (PF) [13]. Thus, the ability to produce force rapidly is crucial [15]. Similarly, peak power output has been shown to be the most distinguishing characteristic among elite level weightlifters [16]. Thus, tracking force-related characteristics for weightlifters is advantageous, as these characteristics underpin performance in competition.

Weightlifting is a sport that requires coordination [17], strength [15], and explosiveness [14,15]. Monitoring these qualities over the course of training can enhance performance. Through athlete monitoring, the present study attempted to: (1) evaluate how block periodization training and the prescribed variable manipulation (e.g., volume load) was reflected in alterations in easily measured performance variables that underpin weightlifting performance; (2) assess alterations in weightlifting performance and; (3) identify what measure(s) provide a better “view” of the adaptation process as it unfolded with these athletes.

## 2. Materials and Methods

Detailed monitoring of the training program by systematic and periodic performance measurements was undertaken across several phases of training. Typically, these measurements were obtained at the end of a block of training to ensure the greatest saturation response from the stimuli(us). Daily monitoring required diligent recording of all of the work that the lifters actually performed in training. The performance variables selected for longitudinal monitoring were force-related variables that underpin weightlifting performance. 

This study was a longitudinal investigation consisting of two distinct testing procedures: (1) a laboratory protocol and; (2) an evaluation of weightlifting performance. The laboratory protocol consisted of six laboratory sessions involving: body mass, body composition, strength, strength-related characteristics, power, and power-related characteristics. The second protocol involved measuring weightlifting performance (snatch and clean-and-jerk) in USA Weightlifting sanctioned competitions. Additionally, daily training data were collected from all athletes at all training sessions. All training session were closely supervised by coaches.

### 2.1. Athletes

Seven trained and competitive weightlifters participated in the study (Table 1). Their weightlifting accomplishments, training ages, and weight classes varied (females: snatch = 55.3 ± 6.4, clean and jerk = 69 ± 8.5; males = snatch = 106.5 ± 31.8, clean and jerk 132 ± 31.8). Six of the seven were national level and included three U.S. Senior National Championship qualifiers, one American Open qualifier, two National Collegiate Championship qualifiers, and one regional level weightlifter. All of the subjects were considered well past the period of initial adaptations and thus for the present study, large magnitudes of performance improvement were not expected.

All of the data consisted of monitoring information collected over the course of 20 weeks by weightlifting coaches and sport scientists of the East Tennessee State University (ETSU) Designated Olympic Training Site for weightlifting as part of an ongoing athlete monitoring program. The training took place in the Exercise and Sport Science Laboratory weight room on the campus of East Tennessee State University. This athlete monitoring study was a collaborative effort between the coaches and sport scientists at ETSU and transpired under “real-life” conditions. During this period, the lifters continued to be supervised by their coaches (USAW certified Olympic Training Site coaches) and the training prescription was written with the intent of best preparing the lifters to attain their performance goals as dictated by the competitive calendar (annual plan). The study was conducted with ETSU IRB approval. 

### 2.2. Timeline

The training study began after initial weightlifting performance measurements (week 0). Subsequent weightlifting performance measurements were made three other times during the study (weeks 7, 11, 20) within pre-planned periods of expected peak performance (Table 2). These measurements followed standard competition guidelines (usaweightlifting.org) and took place on the Saturday of the designated week. In addition to daily monitoring, a series of six laboratory testing periods of two days duration were implemented systematically during the study (weeks 1, 6, 10, 13, 17, and 20) in order to measure body mass, body composition, maximum strength, and derivatives of strength-related characteristics. These measurements took place on the Wednesday and Thursday of the designated test weeks. 

### 2.3. The Training Plan

The development of the training program was a collaborative effort and involved input from the weightlifting coach and sport scientists at East Tennessee State University. Multiple scientific sources including reviews of the literature served as its conceptual structural foundation [1,2,11,18,19,20,21]. During normal training periods, the weightlifters trained four times a week, often twice a day. Active rest periods involved reduced training volumes and intensities, and exercises not typically used during normal training blocks (e.g., light overhead squats). 

A sequential block training program was used with a series of four 3–5 week blocks, along with a total of two periods of two week active rest, one interspersed after each performance measurement period across the 20 weeks (weeks 1–2 and 12–13). Exercises were chosen in concert with the set and repetition scheme in an attempt to achieve the goals and objectives of each block (See Table 3). The order of the sequenced phases (stages and blocks) was based on previous literature and coaches’ experience with this form of training [1,2,20,21]. Programs utilizing a similar sequential block approach have been used successfully with advanced weightlifters [15].

Alterations in relative intensities were incorporated into the weekly training plan to produce heavy and light days (Table 4). In the present study, the weightlifters executed most of their target sets (i.e., not warm-up sets) above 70% intensity. In an effort to standardize warm-ups, the subjects were instructed to perform the same number repetitions as prescribed for their target set (e.g., 3 × 5 = 15 reps) for all of their warm-ups, except for their last warm-up set in which they performed two repetitions. The percentages for relative intensities were based on the given set and repetition range, and not the lifter’s one repetition maximum [2,19].

### 2.4. Daily Monitoring

Daily monitoring involved the recording of every repetition executed for volume load (VLwD) calculations (sets × reps × load × vertical displacement). Displacement for each exercise was measured using the V-scope 120^TM^ (Lipman Electronic Engineering Ltd., Ramat Hahayal, Israel). The V-scope allows for instant feedback of the bar path and involves placing a cap on the end of a weightlifting barbell that emits an infra-red beam. Detailed review of the V-scope is provided by Stone et al. [22]. Displacement was included in the VL calculations in order to better estimate mechanical work during different exercises with different displacements [22,23].

### 2.5. Measurements of Underlying Mechanisms (Performance Testing)

**Figure 1:**
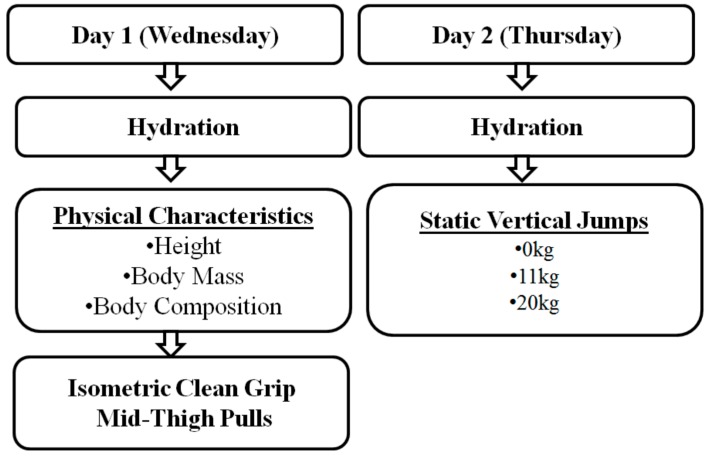
Order of Measurements Executed During a Testing Week.

Tests were scheduled to coincide with major changes in training volume and training foci. Sport scientists worked with coaches to integrate testing into the training process, in order to create the least disturbance to the weightlifters’ training and to maintain the goals of the training blocks (Figure 1). Previous research demonstrated that similar testing protocols have been integrated successfully into an athlete’s training program [11,19]. Testing sessions occurred during the weightlifters morning training session time period. All laboratory testing occurred during the middle of the week.

Hydration preceded both laboratory testing sessions. Hydration status was measured using a refractometer (ATOGO, Tokyo, Japan). If an athlete was found to be dehydrated (urine specific gravity ≥1020), the athlete was required to drink water until the urine specific gravity was <1020 before the testing could be resumed. Testing hydration helps to ensure that the athletes’ hydration status did not influence the tests [24].

### 2.6. Anthropometric

Athlete height was measured using a stadiometer (Detecto, Webb City, MO, USA) and recorded to the nearest centimeter. Body mass was determined using an electronic scale and was measured to the nearest 0.1 kg (BodPOD, COSMED USA, Chicago, IL, USA). Body composition was assessed using plethysmography (BodPOD, COSMED USA, Chicago, IL, USA).

### 2.7. Isometric Mid-Thigh Clean Pull

**Figure 2:**
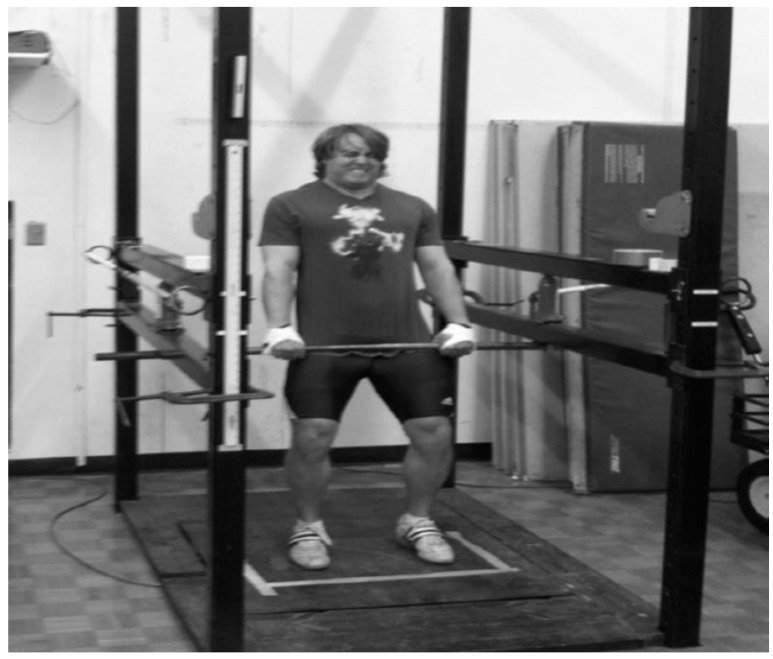
Isometric Mid-Thigh Clean Pull Testing, Note: Photo taken with permission of lifter.

Maximum strength was measured using an isometric mid-thigh clean pull (IMTP), which was performed on a 0.91 m × 0.91 m force plate (Rice Lake Weighing Systems, Rice Lake, WI, USA; 1000 Hz sampling rate) in a custom-designed power rack [15]. The isometric mid-thigh pull was integrated into the weightlifters’ training plan, and measured on Wednesday’s. Wednesday was comprised of mostly weightlifting/pulling movements. The lifters’ hip and knee angles were measured with a hand-held goniometer. Knee angles were set within 125 ± 5° (full extension = 180°, and the hip angle was set at approximately 145°). This position is often referred to as the “power position” and simulates the start of the second pull of a clean (Figure 2) [13,15]. Isometric force was generated when an individual pushed vertically downward on the force plate and pulled up on the immovable bar. Other benefits of the IMTP are that it is relatively quick to test and allows for the measurement of maximal strength while producing much less fatigue compared to dynamic testing (e.g., 1RM back squat). The IMTP power rack and standard pulling position were established based on previously published data [25,26].

Prior to performing the IMTP, athletes followed a standard warm-up consisting of 25 jumping jacks, followed by three sets of five repetitions with dynamic mid-thigh pulls at 30% of their previously established 1RM power clean after one set with a 20 kg barbell. Two warm-up trials of IMTP were performed (self-determined 50% and 75% effort) in the customized power rack (Figure 2). The athletes’ hands were attached to the IMTP barbell using weightlifting straps and standard athletic tape to prevent their hands from moving and to ensure that the athletes could perform a maximal pull regardless of hand grip strength. The start of the maximum effort pulls began with an oral “three, two, one, pull!” countdown. Two maximum efforts were recorded. Sands and Stone [27] note that for monitoring data using the mean of two trials can reduce inherent measurement error, allowing for a better picture of their current training state. A custom-made analysis program written in LabView (National Instruments Co., Austin, TX, USA) was used to quantify the isometric peak force (IPF) and isometric rate of force development (IRFD) during the first 200 ms of each pull. 

### 2.8. Static Vertical Jumps

**Figure 3:**
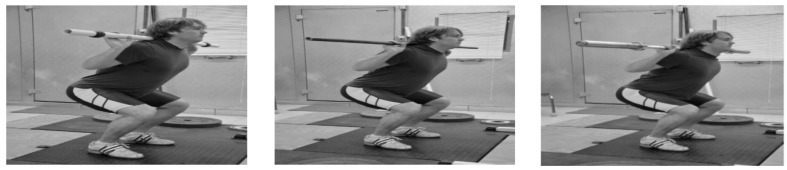
Static Jump Testing with 0 kg, 11 kg, and 20 kg.

The second day of the testing protocol involved measurements of loaded static vertical jumps (0 kg, 11 kg, and 20 kg static jumps), and replaced the weightlifters’ typical Thursday morning squat training session. The SJs were executed with the athletes placing a PVC pipe on their shoulders for the 0 kg load to normalize technique by eliminating an arm swing, while the 11 kg and 20 kg jumps were executed barbells with masses of 11 and 20 kg (Figure 3). The athlete held a 90° knee angle (measured with a hand goniometer) and jumped straight up without a countermovement. The warm-up and verbal command was the same as in the IMTP test except the verbal command, “jump” replaced “pull”. Jump height (JH) and peak power (PP) were calculated for each jump using a custom-made analysis program written in LabView software. Jump height was derived from net impulse. Net impulse was quantified after system weight in Newtons was subtracted from a force-time curve for each jump. Peak power was determined as the maximal value obtained during the jump. Two trials were recorded and averaged for each load.

### 2.9. The Expected General Adaptation Trend

The lifters’ first laboratory testing session (week 1) took place during a period of active rest that immediately followed a weightlifting meet. The weightlifting meet was preceded by a 3 week taper period focused on peaking with the goal of elevating preparedness for a competition. Thus, baseline measures were obtained during a period of low fatigue. Testing period two (week 6) followed the highest volume loads of the study with a focus on increasing work capacity. Certain performance variables appear to be more sensitive to fatigue and the emphasis/de-emphasis of the current training block than others [4]. Issurin [4] and Stone et al. [28] indicate that RFD and high-velocity-related variables seem to be less stable than high-force variables (e.g., PF). Thus, during periods of relatively high volume, when more fatigue is accumulated, one may expect a greater decrease in RFD and speed-related variables. 

Testing period three (week 10) took place during the second week of a taper, which was preceded by one week of planned overreaching (5 sets of 5). Planned overreaching [1] involves a substantial increase in training volume over a short time period (1–2 weeks), potentially pushing the athlete into functionally overreached state. The increased volume is then followed by a return to normal training, which is often followed by a taper (as in blocks 2 and 6 of this study). The purpose of planned overreaching is to potentially elicit additional adaptation through the increased volume and to dissipate accumulative fatigue and achieve increased preparedness with the return to normal volumes and a taper. The measurements took place on week two of a three week taper. Thus, it is possible that enhancements from the taper may not have been completely detected because fitness characteristics and preparedness may have been rising. A correctly designed and implemented taper in volume load can result in elevated preparedness and an increase in performance capabilities as fatigue dissipates and fitness remains acutely elevated, allowing athletes to best express their acquired adaptations [11].

Testing period four (week 13) occurred during the active rest phase which followed the taper. A decrease in performance capabilities would be expected because these measurements were made after 5 weeks of de-loading (taper + active rest) (10). Testing period five (week 17) took place during the third week of a three week strength/power block emphasizing primarily maximal strength. An increase in performance from the previous testing session(s) would be expected due to the de-loading that occurred during the previous phase (testing period 4). Lastly, testing period six involved a strength/power block in which peak power was emphasized. While volume load diminished after the first week of the block (5 × 5), it was not the considered a true taper as VLwD was not reduced to a great enough extent for the coaches to consider it a “true taper” and simply a workload reduction. Testing period three involved the removal of the back squat for weeks nine through eleven, in an effort to further reduce fatigue in the back and lower body musculature. For comparison, the average VLwDs were statistically different (*p* < 0.001) with the taper block presenting the lower VLwD. Based on volume load alterations, one would expect an increase in performance from testing point five to testing point six. 

### 2.10. Statistical Analyses

Conventional inferential statistical (CIS) tools will often not differentiate performance characteristics of athletes or treatments in athletes, particularly advanced athletes [29]. Furthermore, CIS require a sample that is often not possible with advanced athletes, simply because there are not many of them. Thus, subtle, but sport meaningful, alterations in performance are often undetected by conventional statistics [29,30]. Thus, the trade-off of low statistical power in a study may be worth the potential for gaining pertinent information dealing with well-trained athletes. 

Due to the limited sample size, the present investigation attempted to serve as a resource to help form a new hypothesis by describing changes in weightlifting performance and its underlying variables over the course of 20 weeks with limited use of inferential statistics. Buchheit [30] recently published an editorial on “sport science reporting in the real world” noting the benefits of magnitude based inferences (MBI). Thus, MBI based techniques such as percent change, effect size (Cohen’s d), and graphical examinations were utilized [29,30]. Furthermore, an ANOVA trend analysis was performed, where appropriate, in order to investigate the probability of a trend in changes in measures. Additionally, Pearson product-moment correlation coefficients were used to help examine relationships between performance measures and laboratory measures. All inferential statistics and descriptive statistics were calculated using SPSS 19.0 (IBM Corp., Armonk, NY, USA). For inferential statistics, the critical alpha was set at ≤0.05.

## 3. Results

Males (*n* = 4) and females (*n* = 3) were analyzed separately. The reason for this separation was due to the degree of similarity within the males and females (e.g., body mass, strength related variables), but not between the two groups.

### 3.1. Volume Load

**Figure 4:**
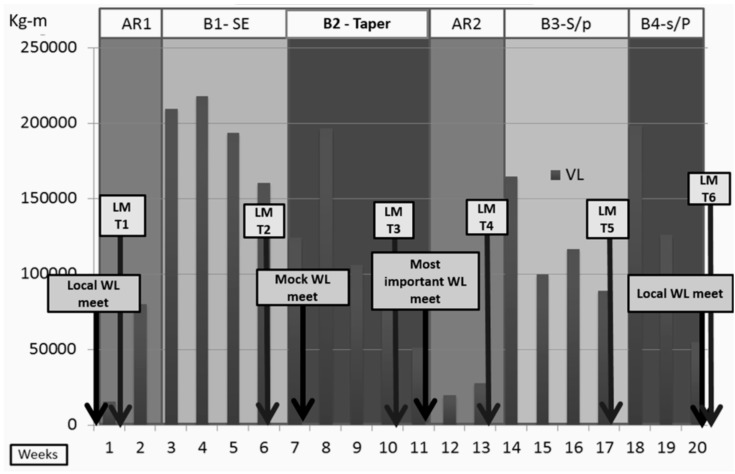
The Weightlifters’ Weekly Training Volume Load (kg × Displacement) Across the 20 Weeks.

Due to strength differences, the males experienced greater absolute VLwD than the females (approximately 1.6 times greater). However, the relative changes in VLwD from one training block to the next were equivalent for males and females (Figure 4) (percent VLd change from block 1 to block 2: females = −43%, males = −40%, percent change from block 2 to block 3: females = −76%, males = −77%, percent change from block 3 to block 4: females = 1%, males = 0.3%).

### 3.2. Body Composition

Minor changes (females <1%, males <2%) were observed in body mass at all six testing points during the study (Table 5). Additionally, all of the subjects “made weight” for all three of the competitions. Male lifters appear to have experienced a somewhat greater change in body fat percentage and fat-free mass than female lifters. Due to the minor change in body mass, no laboratory-measured variables were adjusted for body mass differences among the lifters.

### 3.3. Weightlifting Performance

Correlations were assessed between the laboratory measures and weightlifting performance. The laboratory measures used for the correlations were measured within a week of weightlifting performance measurements (T1–T6). Based on these correlations, as expected, weightlifters who were stronger, more explosive, and more powerful produced greater totals (IPF = 0.72–0.93, IRFD = 0.62–0.76, PP 0 kg = 0.67–0.97, PP 11 kg = 0.73–0.98, PP 20 kg = 0.92–0.98, JH 0 kg = 0.62–0.71, JH 11 kg = 0.71–0.76, JH 20 kg = 0.63–0.82). Additionally correlations (≥0.67) between PF and other laboratory measures indicate that stronger lifters exhibit greater RFD and PPs.

**Figure 5:**
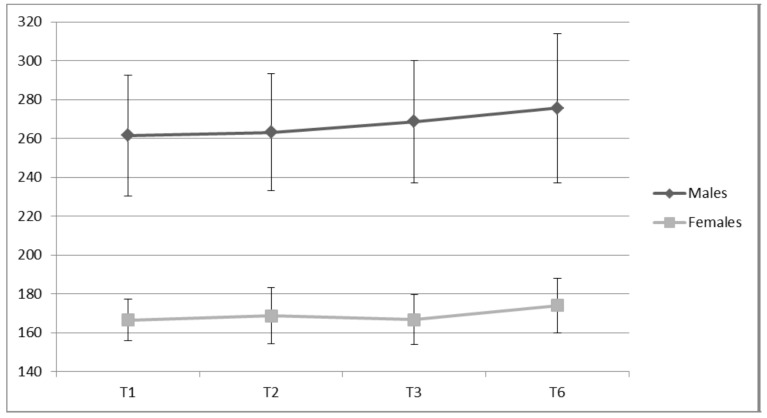
Weightlifting Performance (Sinclair Total in kg) for Males and Females.

The Sinclair formula is a polynomial equation specifically used for weightlifters as a method of obviating body mass differences in weightlifting totals [15]. Both the males and females displayed increases in weightlifting Sinclair performance across the 20 weeks (Figure 5). Laboratory measures took place within one week of assessment of weightlifting performance (meet scenario and conditions) at T1, T2, T3, and T6. This immediacy allowed for comparisons to be drawn between changes in performance variables and changes in weightlifting performance.

### 3.4. Isometric Mid-Thigh Pull

**Figure 6:**
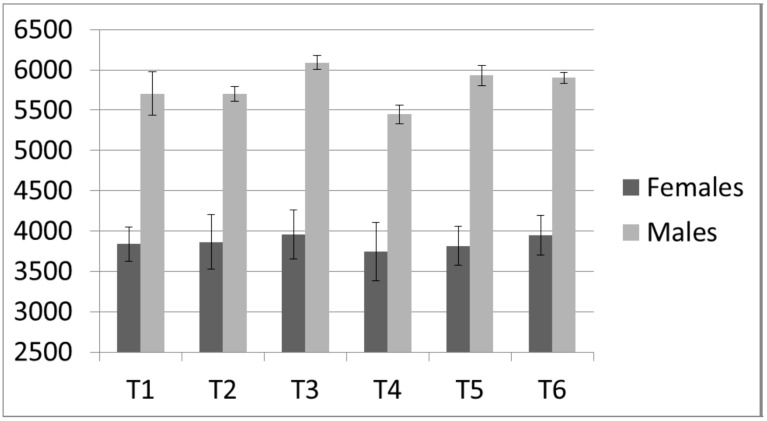
Peak Force from the Isometric Mid-thigh Clean Pull.

**Figure 7:**
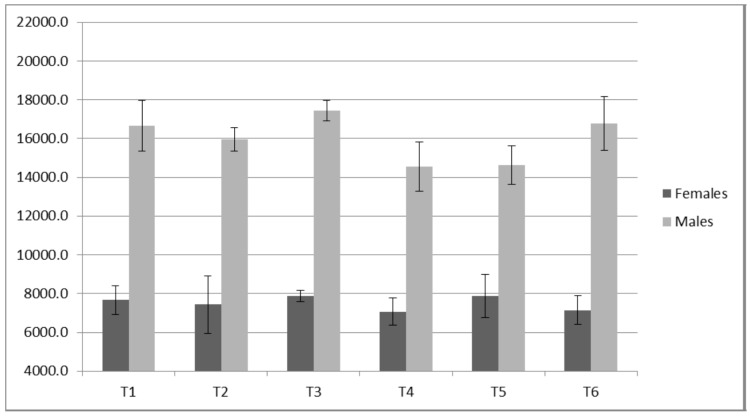
Rate of Force Development from the Isometric Mid-thigh Clean Pull.

Both PF (ICC = 0.98) and RFD (ICC = 0.93) displayed high reliabilities. No statistical (*p* value) significance was observed from the ANOVA trend analysis. From T1 to T2, the females and males PF displayed little change (<1%) (Table 6, Table 7 and Figure 6), while RFD showed a downward trend (females = −3%, males = −4.2%) (Table 6, Table 7 and Figure 7). The associated effect sizes were small (Table 7). From T2 to T3, both PF and RFD showed a positive trend, with RFD showing the greatest percent change (females = 5.88%, males = 9.2%). Additionally, the males expressed a strong effect size for RFD (d = 1.13) (Table 7). While PF did not show an upward trend in the manner that RFD did, PF showed a peak at T3 compared to all other time points (Figure 6 and Figure 7). 

The largest downward trend during the study was noted from T3 to T4 (females PF = −5.29%, females RFD = −10.14%, males PF = −10.53%, males RFD = −16.43%), with large effect sizes being observed for RFD among both females (d = 0.7) and males (d = 1.28), and PF for the males (d = 2.89). The magnitude of fluctuations in PF across the entire study was within 100 N for females. From T4 to T5, the males demonstrated the largest upward trend for PF (effect size = 1.83, percent change = 8.88%) while RFD showed the smallest percent change (0.52%). In contrast, the females demonstrated increases in RFD (11.38%) with trivial changes in PF (1.87%). From T5 to T6, males’ PF remained essentially unchanged (−0.05%), while the females PF showed an upward trend by 3.42%, and RFD trended downward by 9.15%, producing the second lowest RFD during the study.

### 3.5. Static Vertical Jumping

**Figure 8:**
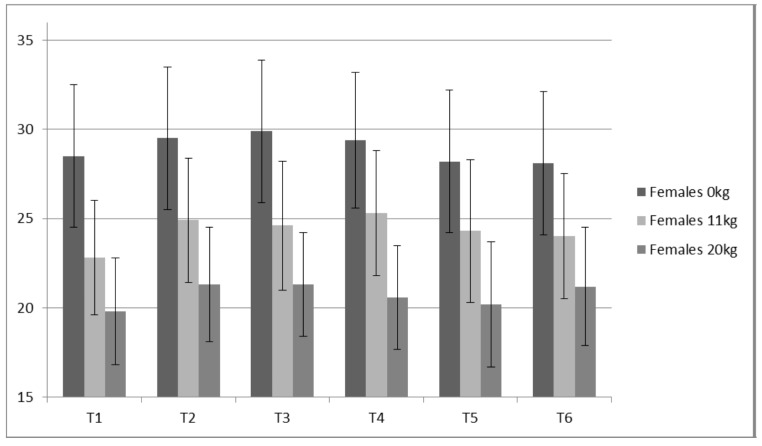
Females Static Jump Height (cm).

**Figure 9:**
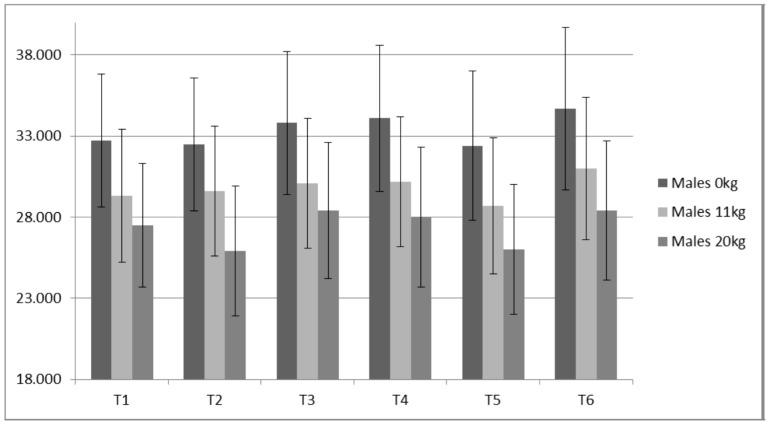
Males Static Jump Height (cm) about here.

**Figure 10:**
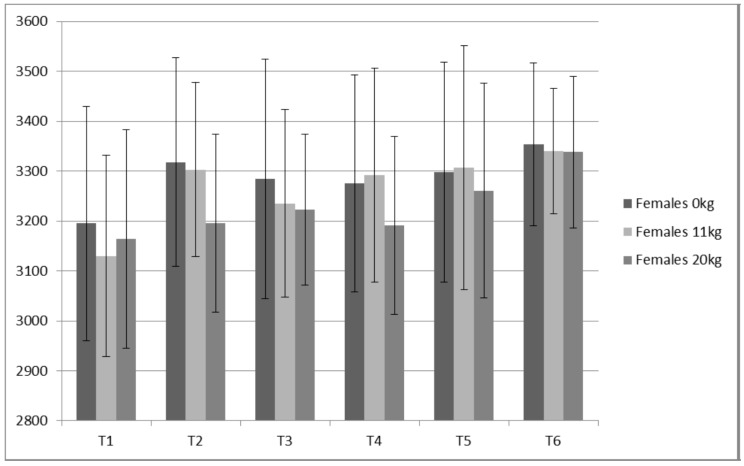
Females Static Jump Peak Power (W).

**Figure 11:**
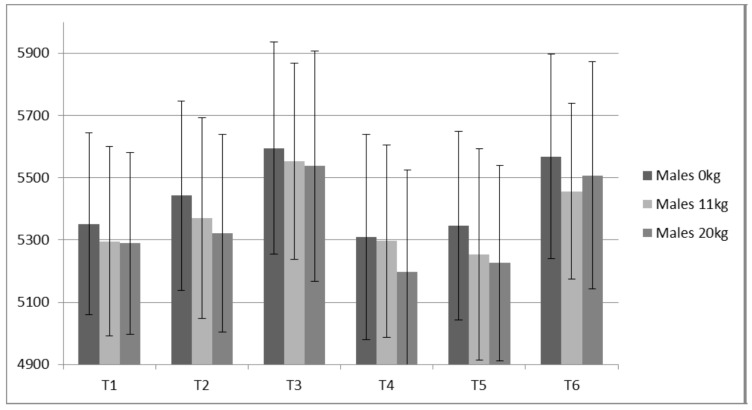
Males Static Jump Peak Power (W).

Both PP (ICC ≥ 0.98) and JH (ICC ≥ 0.93) displayed high reliabilities for all loads measured. Only small changes were observed for JH (Figure 8 and Figure 9) without statistical (*p* value) significance from the ANOVA trend analysis (Table 8). Of all variables measured, PP appeared to present the largest contrast between the males and females (Table 8 and Table 9, Figure 10 and Figure 11). The females demonstrated a statistically significant linear trend (upward) for PP with both 11 kg and 20 kg. For the males, PP fluctuated similarly to IRFD.

## 4. Discussion

The present study aimed to provide a longitudinal observation of weightlifting performance and its association with the underlying variables. While the study’s sample size was limited, it should be noted that the subjects were trained weightlifters and thus adds to the paucity of literature on long-term monitoring among strength-power athletes, particularly weightlifters. Furthermore, the results appear to agree with inferences previously made based on the synthesis of studies of shorter durations and act as a documentation of a weightlifters’ training process from the real-life perspective from daily to weekly to monthly and from competition to competition. 

### 4.1. Body Composition

Negligible changes in body composition are likely due to two reasons: (1) Weightlifting is a weight class sport and thus, the athletes make an effort to stay near their competition weight and; (2) The athletes were well-trained. The minimal chances in the body composition measures provide some evidence that the athletes did not drastically change their diets during the investigation. It also suggests a possibility that performance alterations documented over the course of the study were more attributable to neural adaptations. 

### 4.2. Isometric Mid-Thigh Pull Variables

The IMTP data for males and females followed the expected general adaptation trend. The expectations were based on the specific training focus (e.g., strength-endurance, strength, and speed-strength) for a particular training block and the degree of anticipated fatigue accumulation, which is primarily attributable to volume and secondarily to intensity. The present study involved varying amounts of training volume across the various phases of training consequently leading to changes in performance related variables. Reduction in training volume, to a point, can result in increased strength (e.g., PF), RFD and speed related aspects (e.g., JH). However, reductions in volume load for too long can result in decrements in performance (i.e., detraining).

IMTP data revealed that RFD was quite sensitive to training variable alterations, while changes for PF were much smaller in magnitude. It has been the authors’ observation that PF vs. RFD alterations in weak athletes or untrained subjects are less likely to follow the trends of this study—however, the finding that RFD is much more sensitive to training variable alterations among advanced strength-power athletes has been consistent in our laboratory. The contrasting outcome (PF vs. RFD) has implications from both training and monitoring standpoints. Indeed, this data indicates that the sensitivity of RFD makes it a much better indicator of training strain or accumulated fatigue than PF. Secondarily, the males realized larger absolute and relative changes when compared to the females. 

Based on the training model, improvements in weightlifting performance should have been greatest at T3 and T6. This was especially apparent at T3. Indeed, generally most variables, including weightlifting performance, particularly for the males, did reflect qualitative predictions. When measuring changes towards the end of each training block, it is important to consider not only the most recent phase of training executed, but also preceding blocks as well. Several authors describe an accumulation phenomenon, in which training adaptations, as well as training induced fatigue, converge over time and a brief reduction in volume allows a reduction in fatigue such that the potential to perform well (preparedness) is enhanced [12,31]. It is interesting to note that alterations in performance variables at T6 were, in general, not quite as substantial as at T3. Subtle differences in training variable manipulation may have played a role in these differences in T3 and T6. Measurements at T3 involved a marked taper immediately prior to when the measurements were made. The block that preceded the planned overreaching block (block 2) was a strength-endurance training block (block 1) with a very large volume load. In contrast, while measurements at T6 were also made during a reduction in volume load: (1) the reduction was not as substantial as at T3; (2), nor did the block before it involve as high a volume load and; (3) the preceding block (block 2) lasted 4 weeks for T3, but the preceding block (block 4) lasted only 3 weeks for T6. Some evidence indicates that the previous block’s volume loads may be important for a number of reasons. First, a large reduction in volume load from one block to the next has been proposed to eventually elevate performance capabilities in well-trained athletes [12,31]. Second, planned overreaching, as in block 2 (week 8), may precipitate adaptations during the high volume phase, provided the intensity is large enough [12,31]. Third, a supercompensation effect may occur as a result of diminished fatigue (fitness- paradigm) and/or further physiological (and psychological) alterations such as an increase in type IIx fibers [31]. Fourth, overreaching induced adaptation and subsequent recovery may not have been as complete in a 3 week period (block 4) as a 4 week period (block 2). 

### 4.3. Static Vertical Jump Data

For the males, based on a graphical representation and comparisons between jump height, the training prescription and the IMTP variables, 0 kg and 11 kg, appear to not delineate resultant preparedness as well as the 20 kg. This is based on the 20 kg more closely “matching” the expected trends observed for the IMTP variables, particularly RFD. A good example of this is time point 4 in which IMTP variables were the lowest of the study as the lifters had undergone five weeks of de-loading. Jump heights with 0 kg and 11 kg were not depressed, while jump heights with 20 kg were (Figure 9). Thus, loaded jumps may be a more sensitive monitoring tool.

### 4.4. Manipulating Training Stress

Based on IMTP data for males and females and the peak power data for the males, preparedness appears to have been highest during realization blocks, as predicted. A properly designed and implemented taper can result in elevated preparedness and increase in performance capabilities [1,11]. A strong relationship exists between the amount of work (volume load) executed and the resultant acute and accumulated fatigue [32]. Fatigue can mask a weightlifter’s ability to express various fitness characteristics [1,32]. However, fatigue, even relatively high levels of fatigue, appears to be a necessary part of the training process [32]. Thus, managing fatigue with the objective of maximizing training adaptations while avoiding overtraining requires an understanding of the relationship between fatigue and various amounts and types of training volume. In agreement with other researchers [1,3,28] RFD is less stable than maximum strength (e.g., PF). As RFD is strongly influenced by the nervous system, it’s sensitivity may be a result of the nervous system alterations that have less effect on maximum strength (PF). It is also possible that the alterations in RFD are at least partially due to alterations in myosin heavy chains and fiber type. High volumes of work can stimulate AMKP kinase substantially and result in a shift from Type IIx toward slower fiber type, thus decreasing RFD [15]. Regardless, increased volumes large enough to result in MHC shifts would likely be accompanied by substantial fatigue.

Potential ramifications exist for the sequencing of training blocks, as maximal strength appears to be more resistant to reduced volume load and thus can be de-emphasized further from a major competition while power and particularly RFD seem to fall off much more quickly and thus should be emphasized closer to a major competition [1,18]. The present data along with this theoretical roadmap for sequencing blocks of training is similar to previous literature on sequenced training utilizing an emphasis for a specific fitness characteristic (or concentrated load) for each block [1,2,3,4]. Additionally, training among advanced athletes that is focused on increasing hypertrophy or maximal strength typically produces greater fatigue than training focused on increasing RFD and velocity.

In summary: this observation highlights several important factors: (1) until recently, little evidence has been presented to substantiate the efficacy of theoretical training models, particularly over a long-term (>12 weeks). Block periodization has theoretical underpinnings, which indicate, at least qualitatively, that the direction of performance can be predicted. The present observation largely substantiates those underpinnings. (2) This study outlines several simple performance oriented monitoring tools (e.g., strength and jump tests) that can be used effectively for tracking alterations among strength power athletes. (3) The tests provide a means for valuable feedback to the coaches both as group means and as individual data (not shown in this study), giving the coaches information on the current state of preparedness of the athletes and an index of whether the athletes are realizing expected direction alterations. 

From a monitoring perspective, because RFD is more sensitive to training volume, it may provide a reasonable estimate of an advanced athlete’s fatigue state. While RFD derived from the IMTP may be a better monitoring metric, not all coaches have access to a force plate. A switch mat that provides rapid feedback for jump height is much cheaper and requires less technical skills. Based on data from the males, static JH data suggests that 20 kg produces a better picture of the accumulated fatigue compared to 0 kg and 11 kg. This is based on the expectation that monitoring can detect changes in performance due to alterations in preparedness (fitness—fatigue). Specifically, decreases in performance can be related to accumulated fatigue associated with a marked increase in training volume. For example, at T2, the subjects had just performed a prolonged high volume period (strength endurance via sets of 10); RFD and JH 20 kg demonstrated the anticipated fatigue response, while JH 0 kg and JH 11 kg seemingly did not. This is not to say that a coach shouldn’t test their athletes unloaded jump heights, but that using unloaded jumps to make estimations of accumulated fatigue, particularly for strong athletes such as the males in the present study, may be more difficult.

## 5. Conclusions

It should be noted that prediction of sport performance based on the theoretical aspects of a training process (e.g., periodization model and programming) is qualitative in nature. For example, in this study, T3 did not represent the time point for the highest weightlifting performance (weightlifting total). While producing the highest total is no doubt the ultimate goal for a weightlifter, evaluating a program solely based on weightlifting totals can be a mistake as weightlifting performance is effected by a multitude of factors such as body mass management, psychological state, competition tactics, travelling, and technical proficiency. Thus, it should be understood that monitoring can help a coach evaluate a training program, allowing them to more systematically direct their athlete’s adaptations and performance in a logical, data-supported manner. Theoretically, this process can better allow the coach to increase their weightlifters “chances” of performing well in competition via elevating preparedness. Appropriate monitoring of preparedness with RFD and/or with loaded vertical jumps may greatly assist the coach in this endeavor.

## Figures and Tables

**Table 1:** Descriptive Weightlifter Data.

	N	Height (cm)	Body Mass (kg)	Age (year)	RT Age (years)	WL Age (years)	Snatch (kg)	Clean and Jerk (kg)
Males	4	175 ± 3.7	97.42 ± 11.6	27.8 ± 3.1	10.5 ± 5.6	6.1 ± 5.1	106.5 ± 31.8	132 ± 31.8
Females	3	166.2 ± 4.6	64.8 ± 2.9	22.8 ± 3.4	5.3 ± 2.5	3.0 ± 1.4	55.3 ± 6.4	69 ± 8.5

**Table 2:** Overview of the Weightlifters Weekly Training and Testing Schedule.

Week	Measurement	Training Foci	Sets & Repetitions
0	WL		
1	LM	Active Rest	3 × 3
2		Active Rest	3 × 3
3		Strength Endurance	3 × 10
4		Strength Endurance	3 × 10
5		Strength Endurance	3 × 10
6	LM	Strength Endurance	3 × 10
7	WL	Basic Strength	3 × 5 (1 × 5)
8		Planned Overreaching	5 × 5
9		Taper/Peaking	3 × 3 (1 × 5)
10	LM	Taper/Peaking	3 × 3 (1 × 5)
11	WL	Taper/Peaking	3 × 2 (1 × 5)
12		Active Rest	3 × 3 (1 × 5)
13	LM	Active Rest	3 × 3 (1 × 5)
14		Planned Overreaching	5 × 5
15		STRENGTH/power	3 × 3 (1 × 5)
16		STRENGTH/power	3 × 3 (1 × 5)
17	LM	STRENGTH/power	3 × 2 (1 × 5)
18		Planned Overreaching	5 × 5
19		Strength/POWER	3 × 3 (1 × 5)
20	WL & LM	Strength/POWER	3 × 2 (1 × 5)

Note: Type of Measurement: WL = weightlifting performance (snatch, clean and jerk), LM = laboratory measurements (force characteristics); (1 × 5) represents a down set at 15–25%

**Table 3:** Exercises for Non-Active Rest Blocks.

Block 1: Weeks 3–6	Block 2: Weeks 7–11	Block 3: Weeks 14–17	Block 4: Weeks 18–20
**Monday/Thursday**	**Monday/Thursday**	**Monday/Thursday**	**Monday/Thursday**
AM	AM	AM	AM
Squats	Squats (drop after 2nd week)	Squats	Squats
PM	PM	PM	PM
Front Squats	Push Press-	Push Press	Push Jerks (front squat 1st rep)
Standing Press	change to Push Jerks on week 3	Jerk Recoveries	Jerk Recoveries
**Wednesday**	**Wednesday**	**Wednesday**	**Wednesday**
AM	AM	AM	AM
CGSS	CGSS	CGSS	CGSS
CGMTP	CG Pulls-Floor	CG Pulls-Floor	CG Pulls-Floor
PM	PM	PM	PM
CGSS (20% less)	CGSS (20% less)	CGSS (20% less)	CGSS (20% less)
CG Pulls-Knee	CG Pulls-Knee	CG Pulls-Knee	CG Pulls-Knee
CGMTP	CGMTP	CGMTP	CGMTP
SLDL	SLDL	SLDL	SLDL
**Saturday**	**Saturday**	**Saturday**	**Saturday**
SGSS	SGSS	SGSS	SGSS
Undulating Snatch 10 × 1	Undulating Snatch 5 × 1	Undulating Snatch 5 × 1	Undulating Snatch 5 × 1
(up to 85% of best on week 4)	(up to 90% of best on week 4)	(up to 85% of best on week 4)	(up to 90% of best on week 2)
SG-SLDL	Undulating Clean and Jerk 5 × 1	Undulating Clean and Jerk 5 × 1	Undulating Clean and Jerk 5 × 1
Lateral raises	(up to 90% of best on week 3)	(up to 80% of best on week 3)	(up to 90% on week 1)
	SG-SLDL	SG-SLDL	SG-SLDL

Note: SG = snatch grip, CG = clean grip, CGSS = clean grip shoulder shrugs, CGMTP = clean grip mid-thigh pull, SLDL= stiff legged deadlifts, SGSS = snatch grip shoulder shrugs.

**Table 4:** Relative Intensities across the 20 Weeks of Training.

Week	Monday	Wednesday	Thursday	Friday	Saturday
1		60–65%		60–65%	
2	60–65%	65–70%		65–70%	
3	75–80%	70–75%	80–85%		80–85%
4	80–85%	70–75%	80–85%		85–90%
5	85–90%	70–75%	75–80%		90–95%
6	75–80%	70–75%	75–80%		WL
7	75–80%	70–75%	80–85%		80–85%
8	80–85%	75–80%	80–85%		85–90%
9	85–90%	75–80%	75–80%		90–95%
10	90–95%	80–85%	70–75%		90–95%
11	75–80%	70–75%	75–80%		WL
12	70–75%	70–75%		75–80%	
13	75–80%	70–75%		70–75%	
14	75–80%	70–75%	80–85%		80–85%
15	80–85%	75–80%	80–85%		85–90%
16	85–90%	75–80%	75–80%		90–95%
17	90–95%	80–85%	70–75%		90–95%
18	75–80%	75–80%	80–85%		80–85%
19	80–85%	75–80%	80–85%		85–90%
20	85–90%	80–85%	70–75%		WL

**Table 5:** Body Composition Alterations across the 20 Weeks.

Variable	T1	T2	T3	T4	T5	T6	%Δ (T1–T6)
Females Bdm (kg)	64.8 ± 3.7	64.7 ± 2.8	64.9 ± 2.9	64.7 ± 3	65.8 ± 4.3	64.8 ± 2.9	0
Females % Fat	16.5 ± 6.3	15.9 ± 4.3	16.1 ± 4.7	15.2 ± 6.4	17.2 ± 6.0	17.0 ± 6.3	3
Females FFM (kg)	53.9 ± 2.6	54.3 ± 0.4	54.4 ± 0.7	54.7 ± 1.6	54.3 ± 0.7	53.7 ± 1.7	−0.4
Males Bdm (kg)	97.4 ± 11.6	100.4 ± 10.9	99.1 ± 10.8	99.4 ± 10.9	100 ± 11.4	99 ± 11.8	1.6
Males % Fat	22.5 ± 10.4	19.7 ± 10.6	20.5 ± 11.9	21 ± 11	21.6 ± 11	20.9 ± 10.9	−7.1
Males FFM (kg)	74.8 ± 7.06	79.9 ± 6.2	78 ± 8	77.6 ± 6.9	77.7 ± 5.6	77.5 ± 6.9	3.6

Note: Bdm = Body Mass, FFM = fat free mass.

**Table 6:** Peak Force and Rate of Force Development from the Isometric Mid-thigh Pull for Females and Males.

	T1	T2	T3	T4	T5	T6	*p* Value
Females PF (N)	3840 ± 440	3865 ± 706	3952 ± 3641	3745 ± 756	3815 ± 502	3946 ± 519	0.403
Females RFD (N∙s^−1^)	7663 ± 1581	7430 ± 3141	7867 ± 600	7069 ± 1476	7873 ± 2352	7152 ± 1580	0.727
Males PF (N)	5705 ± 621	5703 ± 193	6089 ± 178	5448 ± 5448	5932 ± 272	5900 ± 131	0.771
Males RFD (N∙s^−1^)	16,652 ± 3042	15,952 ± 1397	17,427 ± 1209	14,563 ± 2933	14,639 ± 2292	16,772 ± 3210	0.400

*p* values are from the ANOVA trend analyses.

**Table 7:** Percent Change and Effect Size for Isometric Mid-thigh Pull Variables.

MBI	T1–T2	T2–T3	T3–T4	T4–T5	T5–T6	T1–T6
Females PF %Δ	0.65%	2.26%	−5.23%	1.87%	3.42%	2.77%
Females PF d	0.04	0.13	0.29	0.11	0.26	0.22
Males PF %Δ	−0.04%	6.77%	−10.53%	8.88%	−0.05%	3.40%
Males PF d	0.01	2.08	2.89	1.83	0.15	0.43
Females RFD %Δ	−3.00%	5.88%	−10.14%	11.38%	−9.15%	−6.66%
Females RFD d	0.09	0.19	0.7	0.41	0.36	0.32
Males RFD %Δ	−4.20%	9.25%	−16.43%	0.52%	14.57%	0.72%
Males RFD d	0.3	1.13	1.28	0.03	0.76	0.38

MBI = magnitude based inference; %Δ = percent change, d = effect size.

**Table 8:** Static Vertical Jump Data.

	T1	T2	T3	T4	T5	T6	*p* Value
Females 0 kg JH	28.5 ± 3.9	29.5 ± 5.3	29.9 ± 5.4	29.4 ± 4.2	28.2 ± 6.1	28.1 ± 5.6	0.55
Females 11 kg JH	22.8 ± 4.1	24.9 ± 4	24.6 ± 4.6	25.2 ± 3.9	24.3 ± 6	24 ± 3.9	0.223
Females 20 kg JH	19.8 ± 3	21.3 ± 4	21.3 ± 2	20.6 ± 2.6	20.2 ± 5.2	21.2 ± 3.9	0.715
Females 0 kg PP	3195 ± 495	3318 ± 437	3284 ± 505	3275 ± 457	3298 ± 463	3353 ± 340	0.365
Females 11 kg PP	3130 ± 424	3303 ± 365	3235 ± 392	3292 ± 448	3307 ± 512	3340 ± 257	0.029
Females 20 kg PP	3164 ± 496	3196 ± 372	3223 ± 313	3191 ± 371	3261 ± 451	3338 ± 315	0.048
Males 0 kg JH	32.7 ± 7.5	32.5 + 7.2	33.8 + 7.5	34.1 + 8.3	32.4 + 8	34.7 + 9.2	0.572
Males 11 kg JH	29.3 ± 7	29.6 ± 7	30.1 ± 6.7	30.2 ± 6.7	28.7 ± 7.6	31 ± 8	0.197
Males 20 kg JH	27.5 ± 6.7	25.9 ± 7.5	28.4 ± 7.4	28.0 ± 7.8	26 ± 7	28.4 ± 7.7	0.266
Males 0 kg PP	5257 ± 672	5361 ± 698	5560 ± 781	5240 ± 758	5193 ± 698	5536 ± 753	0.466
Males 11 kg PP	5240 ± 697	5273 ± 740	5477 ± 724	5252 ± 709	5159 ± 781	5408 ± 646	0.396
Males 20 kg PP	5261 ± 669	5239 ± 730	5471 ± 849	5216 ± 752	5130 ± 721	5411 ± 840	0.756

Note: JH = jump height, PP = peak power.

**Table 9:** Percent Change and Effect Size for Peak Power at 0 kg, 11 kg, and 20 kg.

MBI	T1–T2	T2–T3	T3–T4	T4–T5	T5–T6	T1–T6
Females PP 0kg %Δ	3.85%	−1.03%	−0.25%	0.69%	1.65%	4.90%
Females PP 0kg d	0.26	0.07	0.02	0.05	0.13	0.37
Males PP 0kg %Δ	1.98%	3.71%	−5.76%	−0.90%	6.60%	5.30%
Males PP 0kg d	0.15	0.27	0.42	0.06	0.47	0.39
Females PP 11kg %Δ	5.52%	−2.05%	1.76%	0.44%	1.00%	6.70%
Females PP 11kg d	0.44	0.18	0.13	0.03	0.08	0.6
Males PP 11kg %Δ	0.63%	3.87%	−4.11%	−1.77%	4.83%	3.20%
Males PP 11kg d	0.05	0.28	0.31	0.12	0.35	0.25
Females PP 20kg %Δ	1.00%	0.86%	−1.00%	2.19%	2.36%	5.50%
Females PP 20kg d	0.07	0.08	0.09	0.17	0.2	0.42
Males PP 20kg %Δ	−0.42%	4.43%	−4.66%	−1.65%	5.48%	2.85%
Males PP 20kg d	0.03	0.29	0.32	0.12	0.36	0.2

MBI = magnitude based inference; %Δ = percent change, d = effect size.
